# Prevalence of neural tube defects among pregnant women in Addis Ababa: a community-based study using prenatal ultrasound examination

**DOI:** 10.1007/s00381-023-05901-8

**Published:** 2023-03-03

**Authors:** Abenezer Tirsit, Daniel Zewdneh, Mahlet Yigeremu, Aga Legese, Bente E. Moen, Rolv T. Lie, Morten Lund-Johansen, Rupavathana Mahesparan

**Affiliations:** 1 Division of Neurosurgery, College of Health Science, Addis Ababa University, Addis Ababa, Ethiopia; 2 Department of Clinical Medicine, University of Bergen, Bergen, Norway; 3 Department of Radiology, College of Health Science, Addis Ababa University, Addis Ababa, Ethiopia; 4 Department of Gynecology and Obstetrics, College of Health Science, Addis Ababa University, Addis Ababa, Ethiopia; 5 Department of Global Public Health and Primary Care, University of Bergen, Bergen, Norway; 6 Centre for Fertility and Health, Norwegian Institute of Public Health, Oslo, Norway; 7 Department of Neurosurgery, Haukeland University Hospital, University of Bergen, Bergen, Norway

**Keywords:** Neural tube defect, Prenatal ultrasound, Ultrasound prevalence, Dysmorphology

## Abstract

**Purpose:**

The primary aim of this study was to estimate the prevalence of NTDs at ultrasound examination in communities of Addis Ababa and secondarily to provide a description of the dysmorphology of the NTD cases.

**Methods:**

We enrolled 958 pregnant women from 20 randomly selected health centers in Addis Ababa during the period from October 1, 2018, to April 30, 2019. Of these 958 women, 891 had an ultrasound examination after enrollment, with a special focus on NTDs. We estimated the prevalence of NTDs and compared it with previously reported hospital-based birth prevalence estimates from Addis Ababa.

**Results:**

Among 891 women, 13 had twin pregnancies. We identified 15 NTD cases among 904 fetuses, corresponding to an ultrasound-based prevalence of 166 per 10,000 (95% CI: 100–274). There were no NTD cases among the 26 twins. Eleven had spina bifida (122 per 10,000, 95% CI: 67–219). Among the 11 fetuses with spina bifida, three had a cervical and one had a thoracolumbar defect while the anatomical site for 7 was not registered. Seven of the 11 spina bifida defects had skin covering, while two of the cervical lesions were uncovered.

**Conclusion:**

We report a high prevalence of NTDs among pregnancies in communities of Addis Ababa based on screening by ultrasound. The prevalence was higher than in previous hospital-based studies in Addis, and the prevalence of spina bifida was particularly high.

**Supplementary Information:**

The online version contains supplementary material available at 10.1007/s00381-023-05901-8.

## Introduction

The World Health Organization (WHO) has estimated that 6% of babies are born with a birth defect, suggesting that millions of babies are born with birth defects per year globally [1]. Nine of ten children born with a serious birth defect are in low- and middle-income countries [2]. Neural tube defects (NTDs) are among the most common categories of major birth defects.

An NTD develops around the 28th day of gestation due to the failure of neurulation or alterations in the morphogenesis or histogenesis of the nervous tissue [3]. A high proportion of affected fetuses die in utero and end in a spontaneous abortion [4], but many cases are live-born. Surgery for neural tube defects (NTD) and hydrocephalus are the most performed neurosurgical procedures in Ethiopia [5].

A global literature review reported NTD prevalence estimates typically ranging from 9 to 22 per 10,000 births, with the lowest in Europe and the highest in the Eastern Mediterranean area [6]. Hospital-based studies from Addis Ababa and Amhara have reported prevalence estimates of NTDs to be between 32 and 128 per 10,000 births [7–10]. Still, the ascertainment of cases may have been incomplete in these hospital-based studies. Home deliveries are common in Ethiopia, and these hospital-based data only represent a selection of births from the population. In a retrospective study from Addis Ababa on surgically treated NTDs [11], only 2% of the cases were diagnosed prenatally.

The aim of the present study was to investigate prospectively the prevalence of intrauterine NTD among pregnant women in the general population of Addis Ababa, to describe the dysmorphology of the cases, and to compare the figures with previously reported hospital-based prevalence estimates from Addis Ababa.

## Material and methods

We designed a prospective cohort study by recruiting pregnant women at selected health centers in Addis Ababa. Within 1–2 weeks after recruitment, the women underwent obstetric ultrasound evaluation at the university hospital.

### Selection of health centers

**Fig. 1:**
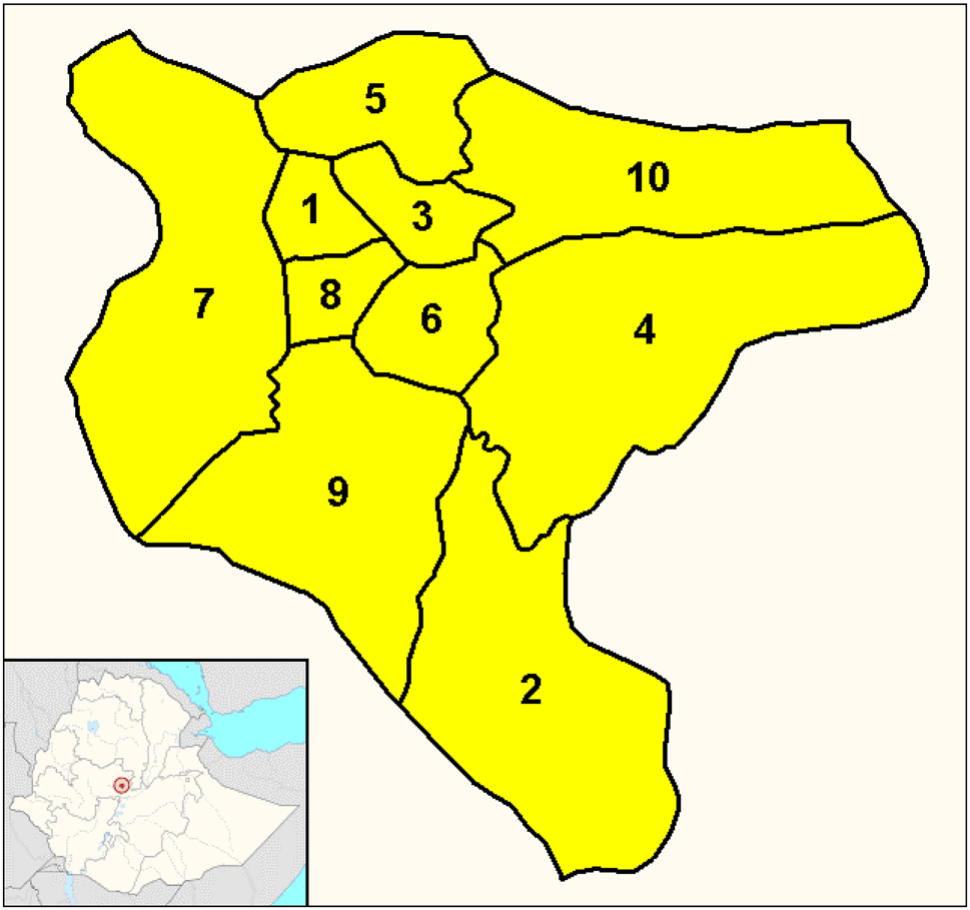
Map over the sub-cities in Addis Ababa, including a map over Ethiopia down to the left, with Addis Ababa marked out. https://commons.wikimedia.org/wiki/File:Addis_Ababa_%28district_map%29.png

With its estimated population size of 115 million (World Bank, 2020, [12]), Ethiopia is the second-most populous country in Africa. Addis Ababa is Ethiopia’s capital and the most urbanized city in the country with an estimated population of 5.5 million. The population is heterogeneous with a substantial number of migrants from rural areas. There are 94 governmental health centers in Addis Ababa. These are located in 10 sub-cities, with 8 to 11 health centers within each (Fig. 1).

To get a representative sample of the population, we selected one health center from each sub-city randomly for the study. Then, we selected a second health center from the same sub-city but with a non-adjacent catchment area and with the availability of antenatal care (ANC) and a number of ANC visits similar to the first. Thus, altogether twenty health centers were involved in recruiting pregnant women for the study. We obtained information about the health centers from the Addis Ababa Health Bureau (AAHB) database.

### Inclusion of pregnant women and ultrasound examination

The study group enrolled pregnant women within 9–22 weeks of gestation into the study during the period between October 1, 2018, and April 30, 2019. Women were invited to participate during their routine ANC appointments. After informed consent, health professionals using a structured questionnaire interviewed the participants about socio-demographics, household, health, pregnancy history, and nutrition. (Table 1).

**Table 1:** Form used at ultrasound for NTD examination

Obstetric ultrasound, 9–12 weeks	
No of intra-uterine pregnancy	
Gestational age using CRL length (mm), (for each fetus if multiple)	
Is there is any cystic mass besides the yolk sac?	
Is transcranial lunacy visible?	
Is the cisterna magna effaced?	
Nuchal translucency distance (mm)	
Obstetric ultrasound between 18 and 22	
Gestational age (for each fetus if multiple)	
Is the supra orbital skull vault formed?	
If no,	Cerebral parenchyma absent Present but disorganized
Is there is any spinal defect in continuity?	
If yes, length of spinal defect (in terms of number of spine)	
If yes to 1608, describe the shape of frontal bone	Concave anterior or lemon shaped Oval Crescent shape
Cerebellar shape	
Herniation of cerebellar tonsil below foramen magnum:	Yes No
Is there is any spinal defect?	Yes No
Is overlying skin	Intact Defective
Level of defect	Cervical Thoracic Thoracolumbar Lumbar Sacral
Gestational age (for each fetus if multiple)	
Is there is any overlying cystic mass in	

We appointed the participants for ultrasound (US) examinations at Addis Ababa University Hospital (Black Lion) within 2 weeks after enrollment in the study. Participants who did not show up for the ultrasound were reminded by telephone. Under the supervision of the senior consultant radiologist, senior residents in radiology scanned the fetuses of pregnant women using Sonoscape SSI-8000 (Via Luigino Tandura, 74–00,128 Rome, Italy). The examiners used a linear 5 MHz probe to magnify superficial structures and a sector 3.5 MHz probe to see details of deeper structures. Gestational age (GA) was assessed by crown-rump length (CRL) during the first trimester and bi-parietal diameter (BPD), femur length (FL), and head circumference (HC) during the second semester. For the earliest weeks after gestation, we diagnosed NTD when there was a cystic mass behind the yolk sac. For the rest of the pregnancy, the diagnosis was made when there was a spinal defect in continuity or signs of encephalocele. We use the same ultrasound machine that is the best available in our setup for all pregnant women. In addition, to reduce variation between measurements, we used pre-stetted protocols for the ultrasound examination and the same senior radiologist was then supervising the examination and checking the findings. We recorded NTD cases using a structured checklist.

### Literature search

We conducted a literature search using PubMed to search for earlier publications about the prevalence of NTDs in Addis Ababa, using the keywords “neural” AND “tube” AND “defects” AND “prevalence” AND “Ethiopia.” In total, the search retrieved 32 publications dated between 2009 and 2022, and of these, we filtered out 19 and kept 20 for abstract review. Following a further full-text evaluation of the articles, we found four papers [7–10] reporting NTDs within the geographical area; however, two of those [8, 9] also included cases outside of Addis Ababa. Therefore, only two of the publications [7] and [10] served as material for comparison with our present data. Supplementary analyses included all four papers (See supplementary figure). We checked the reference lists of these manuscripts to search for additional relevant literature but found none.

The two articles included in the final analysis were hospital-based studies of births with a cross-sectional study [7, 10]. The data extracted from the manuscripts included authors, publication year, study area, period, design and population, sample size, the number of NTDs, and the number of spina bifida cases. The numbers were then combined in a meta-analysis.

### Statistical analysis

Data entry and analyses were conducted using the Statistical Package for Social Science version 21 (SPSS; IBM Corporation, Armonk, New York, USA). We used the metaprop_one-program in STATA v. 17 (StataCorp, College Station, Texas, 77,845, USA) to estimate prevalence with confidence intervals and to perform random effects meta-analyses.

### Ethical approval

We obtained ethical approvals of the study from the IRB of Addis Ababa Health Bureau (Ref. No. AAHB 7029/227), the College of Health Science, Addis Ababa University (Protocol no. 088/17/Surg), and the Regional Committee for Medical and Health Research Ethics, Norway (REK Vest, project no. 103230). All participants received written or oral information and conceded verbally or by signing a consent form.

## Results

We enrolled 958 pregnant women with a mean age of 28 years who conceded to participate in the study (Table 2). Of these, 67 did not have a recorded ultrasound examination and were not included in the study.

**Table 2:** Socio-demographic characteristics of the women

	**NTD in fetus, *n* (%)**	**Not affected, *n* (%)**
Sub-city		
1	5 (33.3%)	241 (27.1%)
2	2 (13.3%)	120 (13.5%)
3	2 (13.3%)	15 (1.7%)
4	0	3 (0.3%)
5	1 (6.7%)	90 (10.1%)
6	2 (13.3%)	125 (14.1%)
7	0	11 (1.2%)
8	0	43 (4.8%)
9	2 (13.3%)	62 (7.0%)
10	0	96 (10.8%)
Unknown	1 (6.7%)	83 (9.3%)
Maternal age( in years)		
Mean, median (SD)	28.7, 30 (4.8)	26.8, 26 (4.5)
Marital status		
Married	14 (93.3%)	839 (94.4%)
Single	1 (6.7%)	37 (4.2%)
Divorce/separated/widowed	0	10 (1.1%)
Unknown	0	3 (0.3%)
Total number	15	889

### Prevalence at ultrasound examination

Altogether, 891 out of 958 conceding participants (93%) underwent NTD examination with ultrasound. Thirteen of the pregnancies were twin pregnancies. Ultrasound examination identified an NTD in 15 of the 904 fetuses (Table 3), giving an estimated ultrasound NTD prevalence of 166 per 10,000 (95% CI: 101–272). None of the cases were among the twins. Eleven of the cases were spina bifida corresponding to a prevalence of 122 per 10,000, (95% CI: 68–217). Numbers were too small to evaluate differences in prevalence between sub-cities (Table 2).

**Table 3:** Overview of the 15 NTD cases identified at the ultrasound examination

**Case number**	Estimated GA*	**Skin covering**	**Diagnostic ultrasound finding**	**Hydrocephalus**	**Level of the defect**	**Status at birth**
1	16	NR^b^	Spinal defect	NR	Cervical	NR
2	21	Intact	Spinal defect	No	NR	NR
3	19	NR	Spinal defect	No	NR	NR
4	9^a^	NR	Cystic mass behind the yolk sac	NR	NR	NR
5	20	NR	Cystic mass behind the yolk sac	NR	NR	NR
6	20	NR	Spinal defect	No	NR	NR
7	15	NR	Cystic mass behind the yolk sac	No	NR	Abortion
8	21	Intact	Spinal defect	No	NR	NR
9	17	Intact	Spinal defect	No	Thoracolumbar	NR
10	21	Defective	Spinal defect	No	Cervical	NR
11	15	Intact	Cystic mass behind the yolk sac	No	NR	Abortion
12	17	Defective	Spinal defect	No	Cervical	NR
13	18	NR	Spinal defect	No	NR	NR
14	20	NR	Spinal defect	No	NR	NR
15	17	Intact	Spinal defect	No	NR	NR

^a^Based only on CRL

^b^*NR*, Not reported

^*^Gestational age at ultrasound estimated from *BPD* (bi-partial diameter), *FL* (femoral length), and *HC* (head circumference)

### Ultrasound NTD characteristics (Table 3 and Fig. 2)

**Fig. 2:**
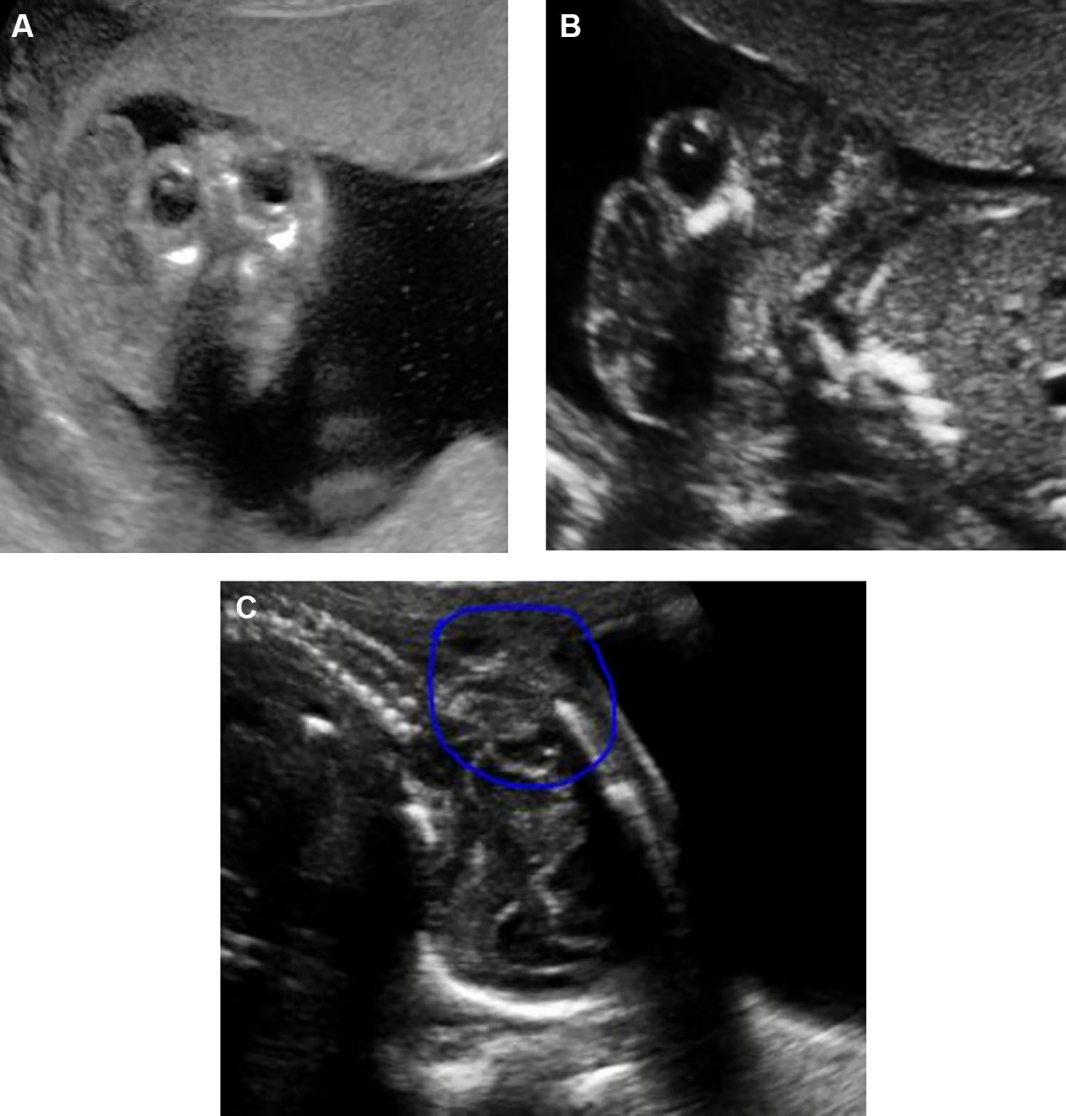
Ultrasound images of fetus with NTDs. **A** Anencephaly case: “Mickey Mouse” head of anencephaly with an absence of cortical tissue as well as an absence of the cranial vault. **B** Encephalocele case: occipital myelomeningocele. **C** Thoracolumbar MMC: spina bifida with myelomeningocele (circled)

A cystic swelling behind the yolk sac was observed in three fetuses at a GA at or above 9 weeks and these fetuses had an absent frontal bone, absent or poorly developed brain parenchyma, and absent ventricles. One fetus had occipital encephalocele; this fetus had a formed supra-orbital skull vault with a concave frontal bone and developed brain parenchyma. A spinal defect in continuity was observed in the remaining 11 fetuses. The mean (median) gestational age (GA) for the 15 fetuses was 16 (17) weeks, range of 15–21.

Among the 11 fetuses with spinal defects, the anatomic level of the defect was registered for only four, three had cervical and one had a thoracolumbar defect. The status of the overlying skin was registered in 11 lesions; there was skin defect in two lesions at the cervical level and intact skin in the nine others.

The size of the lateral ventricles (atrium) at the level of the thalamus was measured in 9 of the 12 fetuses with spina bifida or encephalocele. We found that the mean (median) size of the atrium of the lateral ventricle was 6.26 mm (6.7) on the left side and 6.5 mm (7.1) on the right. Nine of the fetuses had a slit-like third ventricle. One fetus had round posterior lateral ventricles horns, the other eight had pointed posterior horns.

The status of the cerebral cortex was reported in 12 fetuses with NTD, and in 10 fetuses, the cerebral cortex was reported as normal, and the mean (median) cerebellar diameter was 16.5 mm (17 mm).

Of the twelve fetuses with NTDs, 11 fetuses had normal cisterna magna, with no cerebellar tonsil herniation below the foramen magnum, or a sign of syringomyelia. One of the fetuses with anencephaly had an ultrasound picture of the “Mickey Mouse” head and one of the fetus with encephalocele had occipital myelomeningocele (Fig. 2).

### Comparison with previous reports

**Fig. 3:**
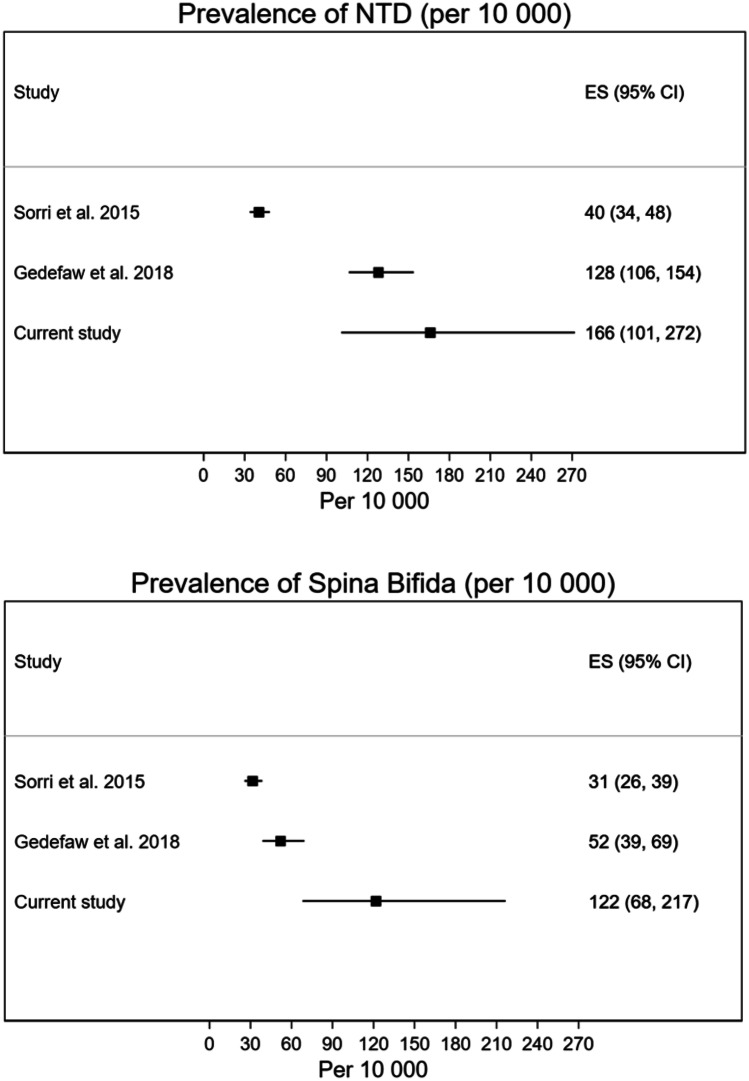
Prevalence of NTD (upper panel) and spina bifida (lower panel) in Addis Ababa from two previous studies and our current study

The two previous studies of NTD prevalence are included in Fig. 3 [7, 10]. They were conducted in public hospitals with 24-h obstetrics and pediatrics services in Addis Ababa. Since some NTD cases result in an abortion or fetal loss, an ultrasound-based prevalence of NTDs in a specific population is likely to be slightly larger than the birth prevalence. The largest study by Sorri and Mesfin [7] reported a clearly lower birth prevalence of NTDs, while our current study of the ultrasound-based prevalence was more similar to the prevalence estimate of Gedefaw et al. [10], which also included abortions of NTD-fetuses. The prevalence of spina bifida from our study appears to be higher than the birth prevalence reported in the two previous studies. Two studies with data partially from Amhara [8, 9] are included in the Supplementary figure. The most recent one [9] had a prevalence estimate of NTDs that were closer to our study and the study by Gedefaw et al. [10].

## Discussion

NTDs represent a substantial health burden in Ethiopia [8, 13, 14]. A systematic meta-analysis from different countries in Africa revealed a pooled birth prevalence of 21 per 10,000 births in Africa and suggested that Ethiopia was one of the countries with the highest prevalence of NTD in Africa [15]. In contrast, a systematic review of data from 75 countries with different health systems and economic developmental levels found a median prevalence of NTD at the birth of 1.1 per 10,000 births [6]. In general, most studies assess children after birth and are hospital-based. Home deliveries are still widespread in Ethiopia, and therefore, many cases may be undetected. We are not aware of any previous studies investigating the intrauterine prevalence of NTD prospectively in the general population of Ethiopia [16].

In our study of 891 pregnant women, we estimated the NTD prevalence to be 166 per 10,000, which is higher than previously reported prevalence estimates from Addis Ababa. Our study was different from the previous studies in several ways that may have contributed to the differences.

Firstly, our study identified NTD-affected fetuses at 9–22 weeks of gestation by ultrasound examination of the pregnant women. One of the studies included in our review, estimated a birth prevalence of 63 per 10,000 for NTDs when considering all births after 28 weeks of gestation, and a prevalence of 128 per 10,000 when induced abortions due to NTDs from 12 weeks of gestation were included [10]. Some cases that got subsequently aborted were included in our study (Table 1). Fetuses with NTDs that were spontaneously aborted probably would have resulted in lower birth prevalence among participants in our study.

Secondly, several of the defects detected in our study were skin covered. Spina bifida was the most common type of NTD in our study (11 of the 15). Seven of those had skin-covered spina bifida suggesting that these fetuses had closed defects or spina bifida occulta, which could be the most common type of NTD in this population. The ultrasound examinations in this study were conducted focusing specifically on detecting NTDs and revealed a high prevalence of NTDs when including those lesions with skin covering. This may suggest that there are more children in the community affected with spina bifida occulta than what is known currently and that we are not yet aware of the full burden of NTDs in this population. Other studies that we reviewed did not include spina bifida occulta [10, 11, 14, 17, 18].

Finally, our study was community-based with the recruitment of pregnant women from the general population. The role of selection in previous studies is difficult to assess. Women with better knowledge and resources might have selected hospitals for delivery in Ethiopia, they may have better living circumstances and thereby a lesser risk of NTD due to malnutrition. This may be another explanation for the lower prevalence of NTDs in previous hospital-based studies.

The population of Addis Ababa includes a heterogeneous mixture of inhabitants from across Ethiopia. Reports from different regions in Ethiopia have shown that the NTD prevalence may vary between regions [9, 13, 14, 17, 18]. This might be due to both genetic and environmental factors in the different regions, and this information is difficult to interpret. The prevalence of NTDs may vary between different sub-cities of Addis Ababa, but our numbers were too small for statistical comparison.

Our study showed that the number of pregnant women who came to antenatal care varied greatly between the sub-cities. We do not know how many pregnant women who did not show up for antenatal care from each sub-city during the study period, so the actual intrauterine prevalence of NTDs in the population may differ from what we found. One can speculate that the real figures for NTDs might be even higher.

A meta-analysis and systemic review that included also other parts of Ethiopia showed a pooled NTD and spina bifida prevalence of 63 and 41 per 10,000, respectively [13]. Spina bifida was the most common type of NTD in our data. Most reports, however, show anencephaly to be far more common than spina bifida. [18, 20] Another hospital-based study reported an NTD prevalence at birth of 50 per 10,000 in the southwestern part of Ethiopia [20]. A recent hospital-based cross-sectional study from the Tigray region of Ethiopia estimated a high overall prevalence of NTD of 131 per 10,000 births of which 23% were live-born and 77% stillborn [14].

The ultrasound equipment used is of the best available in Ethiopia; however, the resolution is not up to what is achieved using top-notch equipment, thus implying a risk of occasional false negative examinations. In addition, even though a structured checklist was used during the ultrasound examination, data were often incompletely reported.

During embryogenesis, failure of neural tube closure cranially results in anencephaly, and caudal failure results in spina bifida [3]**.** The anatomic variations seen in spina bifida occur early in development. Ultrasound classifies spina bifida occulta as a disruption involving only bony elements, and spina bifida cystica as a saccular defect involving neural elements [19].

During ultrasound imaging, associated brain and cranial malformations with NTDs can be seen, such as flattened frontal bones, obliteration of the cisterna magna, a banana-shaped cerebellum, and ventriculomegaly [19]. Although cranial malformations are commonly associated with NTDs, we did not find cisterna magna obliteration or cerebellar tonsil herniation below the foramen magnum in any of the fetuses with spina bifida or encephalocele. This could provide some insights into the severity of the defects present in most of the fetuses in our study; probably most had a milder form of dysraphism or skin closed defects in second-trimester pregnancy. Another plausible explanation could be the quality of ultrasound examinations that did not render to detect minor changes.

The sample size of this study was limited but still included a substantial number of NTD cases. Of all the pregnant women recruited into the study, 7% did not undergo an ultrasound examination and were lost to follow-up. It is difficult to know how much this could have influenced the results. However, that we were able to perform ultrasound among so many pregnant women, and that the study was prospective are still clear strengths of the study.

## Conclusion

This study based on an ultrasound examination of a community-based sample of pregnant women estimated a higher prevalence of NTDs than any previous study from Addis Ababa and other parts of Ethiopia and a higher prevalence than in other studies globally. The prevalence of spina bifida was particularly high. The reason for this high prevalence of NTD in Ethiopia should be investigated further with the aim to develop prevention strategies for this disease.

## Supplementary Information

Below is the link to the electronic supplementary material.

Supplementary file1 (DOCX 6455 KB)
