# Supplementary material for: Prevalence of neural tube defects among pregnant women in Addis Ababa: a community-based study using prenatal ultrasound examination
Source: Childs Nerv Syst. 2023 Mar 3;39(9):2423–31. doi: 10.1007/s00381-023-05901-8 (PMC10432327; doi:10.1007/s00381-023-05901-8)

**Supplementary figure. Prevalence of NTD (upper panel) and Spina Bifida (lower panel) in Addis Ababa reported from four previous studies and our current study. The two studies by Taye et al. also included data from Amhara.**

**
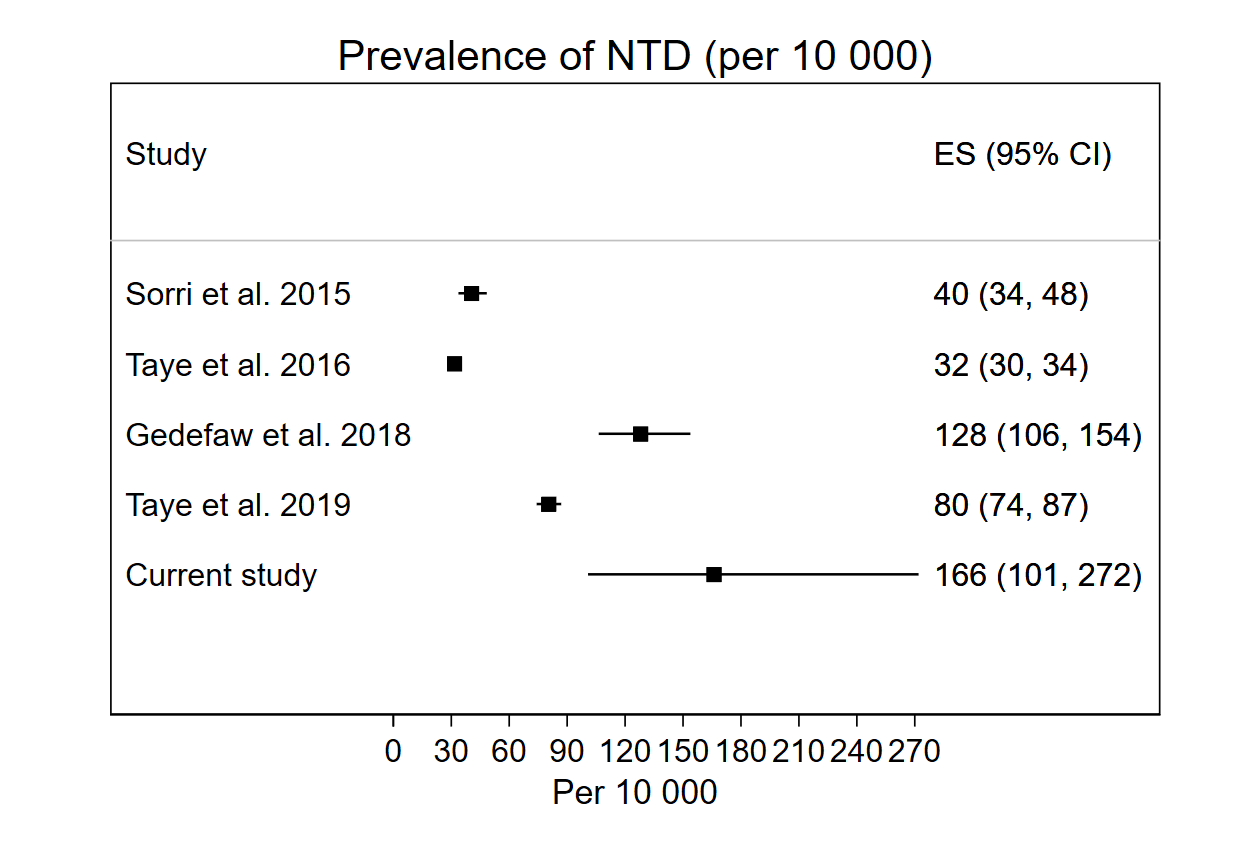
**


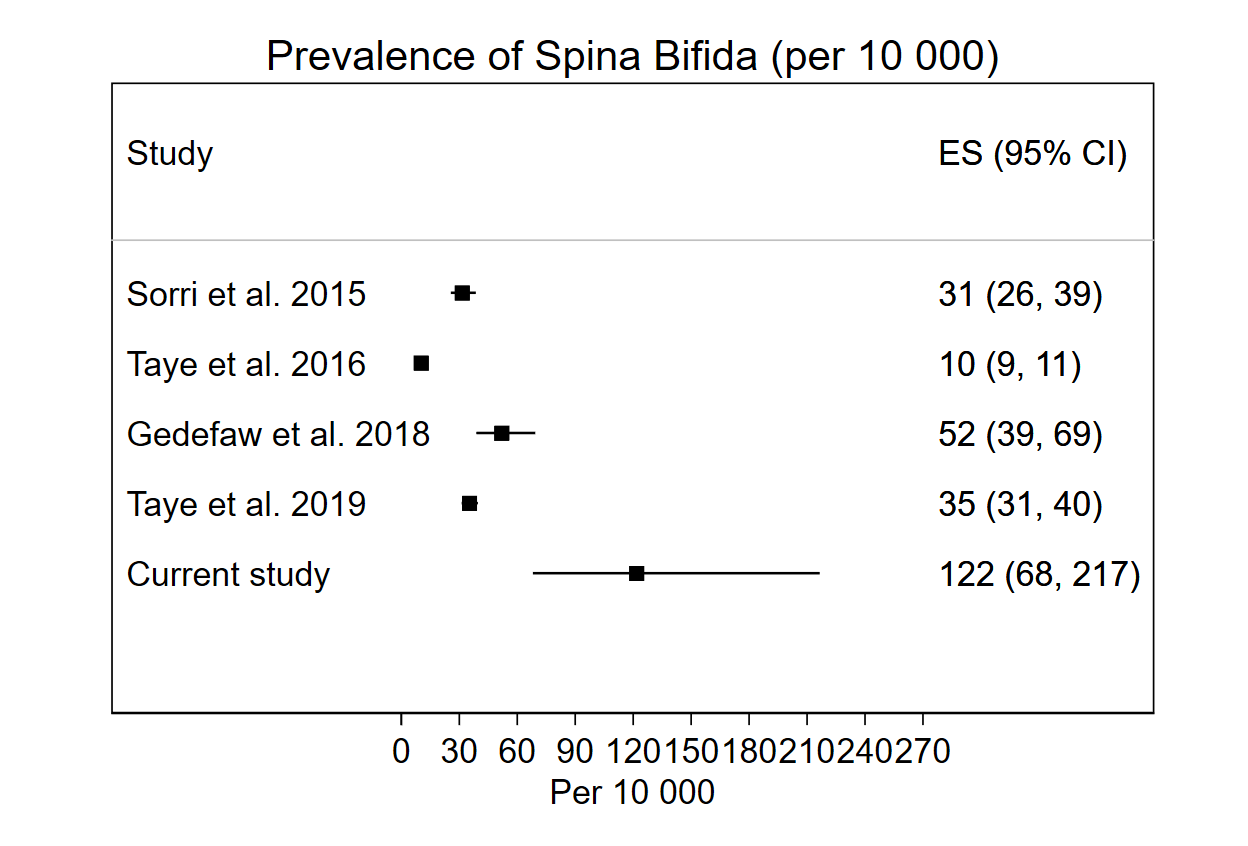

Supplement: Supplementary file 1 — Supplementary file1 (DOCX 6455 KB) [file 381_2023_5901_MOESM1_ESM.docx]
